# Auxin-Glucose Conjugation Protects the Rice (*Oryza sativa* L.) Seedlings Against Hydroxyurea-Induced Phytotoxicity by Activating UDP-Glucosyltransferase Enzyme

**DOI:** 10.3389/fpls.2021.767044

**Published:** 2022-02-16

**Authors:** Vimalraj Kantharaj, Nirmal Kumar Ramasamy, Young-Eun Yoon, Mi Sun Cheong, Young-Nam Kim, Keum-Ah Lee, Vikranth Kumar, Hyeonji Choe, Song Yeob Kim, Hadjer Chohra, Yong Bok Lee

**Affiliations:** ^1^Division of Applied Life Science (BK 21 Four), Gyeongsang National University, Jinju, South Korea; ^2^Department of Biotechnology, PSGR Krishnammal College for Women, Coimbatore, India; ^3^Institute of Agriculture and Life Science (IALS), Gyeongsang National University, Jinju, South Korea; ^4^Department of Smart Agro-Industry, Gyeongsang National University, Jinju, South Korea; ^5^Division of Plant Sciences, University of Missouri, Columbia, MO, United States

**Keywords:** auxin, auxin conjugate, hydroxyurea (HU), reactive oxygen species (ROS), ribonucleotide reductase (RNR), uridine 5′-diphosphate-glucosyltransferase (UGT)

## Abstract

Hydroxyurea (HU) is the replication stress known to carry out cell cycle arrest by inhibiting ribonucleotide reductase (RNR) enzyme upon generating excess hydrogen peroxide (H_2_O_2_) in plants. Phytohormones undergo synergistic and antagonistic interactions with reactive oxygen species (ROS) and redox signaling to protect plants against biotic and abiotic stress. Therefore, in this study, we investigated the protective role of Indole-3-acetic acid (IAA) in mitigating HU-induced toxicity in rice seedlings. The results showed that IAA augmentation improved the growth of the seedlings and biomass production by maintaining photosynthesis metabolism under HU stress. This was associated with reduced H_2_O_2_ and malondialdehyde (MDA) contents and improved antioxidant enzyme [superoxide dismutase (SOD), ascorbate peroxidase (APX), catalase (CAT), and peroxidase (POD)] activity that was significantly affected under HU stress. Furthermore, we showed that the HU stress-induced DNA damage leads to the activation of uridine 5′-diphosphate-glucosyltransferase (UGT), which mediates auxin homeostasis by catalyzing IAA-glucose conjugation in rice. This IAA-glucose conjugation upregulates the RNR, transcription factor 2 (E_2_F_2_), cyclin-dependent kinase (CDK), and cyclin (CYC) genes that are vital for DNA replication and cell division. As a result, perturbed IAA homeostasis significantly enhanced the key phytohormones, such as abscisic acid (ABA), salicylic acid (SA), cytokinin (CTK), and gibberellic acid (GA), that alter plant architecture by improving growth and development. Collectively, our results contribute to a better understanding of the physiological and molecular mechanisms underpinning improved growth following the HU + IAA combination, activated by phytohormone and ROS crosstalk upon hormone conjugation *via* UGT.

## Introduction

Plants, as sessile organisms, are constantly challenged by a wide range of environmental stresses that often cause crop or yield losses. Reactive oxygen species (ROS) is an important signaling molecule that regulates plant growth and development in response to various stresses. Under adverse environmental stress, plants accumulate excess ROS, specifically H_2_O_2_, which results in molecular, biochemical, and physiological damage due to over-oxidation of essential macromolecules such as DNA, protein, and membrane lipids by inhibiting ROS-scavenging enzymes (Das and Roychoudhury, 2014). The interplay between ROS and plant hormones regulates the stress response and drives changes in transcriptomic, metabolic, and proteomic networks that lead to plant acclimation and survival (Bright et al., 2006). In Arabidopsis, ROS directly regulates the auxin activity in an H_2_O_2_-dependent mitogen-activated protein kinase cascade, which alters auxin sensitivity by suppressing the expression of auxin-inducible genes (Xia et al., 2015). Apart from auxin, ROS is also induced by drugs like HU and Bleomycin (BM) and it is also found to affect cell cycle regulation (Vanderauwera et al., 2011). Hydroxyurea (HU) is the DNA damage agent known to carry out cell cycle arrest by inhibiting the activity of ribonucleotide reductase (RNR) enzymes leading to an altered dNTP pool essential for DNA replication and cell cycle progression (Sakano et al., 2001; Cools et al., 2010; Winnicki, 2013). The RNR function is to make deoxyribonucleotides by directly reducing ribonucleotides, which is the rate-limiting step in *de novo* synthesis (Reichard, 1988). The HU directly targets RNR by inhibiting H_2_O_2_ decomposing enzyme catalase. The RNRs are localized in the chloroplast, and the altered activity decreases the cell cycle, chlorophyll biosynthesis, and retarded growth (Juul et al., 2010; Gu et al., 2020).

Glycosylation is a widespread molecular modification in both prokaryotic and eukaryotic species and is critical to maintaining metabolic balance by regulating the signaling molecule and defense compound (Lairson et al., 2008). Glucosyltransferases are responsible for glycosylation which binds active donor sugar moieties to various receptor molecules, including a given class of phytohormones, metabolites, xenobiotics, and pathogen antigens (Coutinho et al., 2003; Bowles et al., 2005). Phytohormones and their conjugations are considered vital due to their crucial hormone homeostasis role in regulating physiological active hormone levels during a plant’s growth and development. Most plant hormone conjugation is not physiologically active, but instead, it is known to participate in the transport, storage, and degradation (Sembdner et al., 1994). Auxins are the plant hormones that are well-known for their regulatory roles in plant growth and development throughout its life cycle, including apical dominance, vascular development, postembryonic organogenesis, plant tropism, fruit development, senescence, and stress response (Sundberg and Østergaard, 2009; Grunewald and Friml, 2010). At the same time, the endogenous auxin level is maintained by various mechanisms, including biosynthesis, degradation, transport, and formation of conjugation (Bajguz and Piotrowska, 2009). Over the decades, several crucial hormone glucosyltransferases that catalyze the transfer of activated sugars to hormones in plants play vital roles in regulating their homeostasis and stress tolerance. For example, rice OsIAGT1 (Liu et al., 2019), OsZOG1 (Shang et al., 2016), Arabidopsis AtJGT1 (Song, 2005), and bean PvABAGT (Palaniyandi et al., 2015) have the glucosylating activity toward IAA, cytokinin (CTK), Jasmonic acid (JA), and abscisic acid (ABA). The *iaglu* was the first cloned IAA glucosyl transferases gene in maize (Szerszen et al., 1994). But their biochemical characterization has been reported by Leźnicki and Bandurski (1988) and Kowalczyk and Bandurski (1991). In rice, *iaglu* was recently characterized to show regulatory activity on seed vigor, root growth, and stress tolerance by integrating multiple hormonal pathways (He et al., 2020; Zhao et al., 2020). The auxin glucosyltransferases UGT84B1, UGT74E2, and UGT74D1 genes are known to encode auxin glucosyltransferases in Arabidopsis (Jackson et al., 2001). However, their glycosylation activity differs between IAA and indole-3-butyric acid (IBA), while UGT84B1displayed higher activity toward IAA, with considerable activity toward IBA and indole-3-pyruvic acid (IPA) (Jackson et al., 2001), UGT74E2, and UGT74D1 chose IBA as their conjugation substrate rather than IAA (Tognetti et al., 2010). In addition, the activity of IAA glucosyltransferases has also been identified in the seeds of pea *(Pisum sativum)* (Jakubowska and Kowalczyk, 2004; Ostrowski et al., 2015). The effect of auxin application on the Arabidopsis roots treated with HU stress displayed a phenotype with a considerable increase in the number of sites of lateral root initiation with respect to control and HU treatment, and the mechanism responsible for the growth-promoting role following HU + NAA treatment remains elusive (Topping and Lindsey, 1997). Based on the available knowledge in literature, the combined treatment of HU and phytohormones (i.e., auxin and cytokinin) in response to the plants still awaits detailed investigation. Thus, studies concentrating on how phytohormones (i.e., IAA) influences the HU stress should be of a broader scope that addresses a set of questions: (1) What is an interaction between the IAA and hydroxyurea? (2) How IAA improves the plant growth under HU stress. And most importantly, what is the mechanism involved in enhancing plant growth upon HU + IAA. Answering these questions is undoubtedly, a long-lasting task, and requires knowledge from multiple aspects like hormone conjugation, phytohormone crosstalk, and ROS signaling pathway.

With this backdrop, the present study was designed to elucidate the possible mechanism involved in the IAA-mediated changes of HU toxicity in retarded rice seedlings. The influence of IAA on HU-induced changes of growth, photosynthesis, DNA damage, cell division, cellular integrity, and antioxidant defense enzymes activities has been investigated. This study provides new insights into physiological and molecular mechanisms underlying enhanced growth upon HU + IAA treatment, triggered by the interplay of phytohormones with ROS signaling, and paved the way for the functional relevance of HU on plant glycosylation studies.

## Materials and Methods

### Plant Genetic Materials, Treatments, and Sampling

The rice cultivar (Dongjin) seeds obtained from the Rural Development Administration (RDA, South Korea) were used in this study. Uniform and healthy seeds were surface-sterilized with 1% (v/v) sodium hypochlorite solution (NaOCl). Then, the seeds were germinated in a Petri dish for 3 days in the dark in a temperature-controlled room at 37°C. After germination, 3-day-old identical seedlings were selected and transferred to an aquaponic growing foam board container with half-strength Murashige and Skoog (½ MS) nutrient medium (Sigma-Aldrich, MO, United States). Then, the plants were grown in MS medium with HU and IAA (alone or combined, for 3 days. Four treatment groups of plants were maintained: control (CK), HU (1 mM), IAA (0.3 μM), and HU (1 mM) + IAA (0.3 μM). Plants were kept in a temperature-controlled growth chamber set at 28°C with 60% relative humidity and a photoperiod of 16/8 h (light/dark). The shoot and root samples were collected from 6-day-old seedlings and stored at –80°C for further experiments.

### Measurement of the Chlorophyll Content and Photosynthetic Rate

The method of Arnon (1949) was followed to measure the total chlorophyll, chlorophyll a, and chlorophyll b contents. Briefly, leaf samples of 200 mg were taken in test tubes and then added to 2 ml of 80% acetone. These samples were incubated at 65°C until the samples became colorless. The chlorophyll contents were read at 645 and 663 nm. The amount of chlorophyll content was calculated and expressed as μg/ml. Photosynthetic efficiency as the quantum yield of photosystem II (i.e., *F*_v_/*F*_m_) was measured as described by Chen et al. (2019) using an ultra-portable modulated chlorophyll fluorometer (Mini-PAM II Photosynthesis Yield Analyzer, WALZ, Germany). The minimal (*F*_0_) and maximal fluorescence yield of the dark-adapted state (Fm) emissions were assessed in the leaves after 20 min of dark adaptation, and the maximum quantum yield of PSII was calculated as Fv/Fm = (Fm–F_0_)/Fm.

### DAB and PI Staining

The H_2_O_2_ superoxide generated inside the cell was observed using DAB staining. The root samples were immersed with DAB-hydrogen chloride (HCl) solution (0.5 mg/ml, pH 3.8) for 30 min with a mild shaking in an incubator at room temperature. The DAB reacted with H_2_O_2_ to develop the brown product in roots, which was further observed and captured using a light microscope. The cell death of the roots of the rice was observed using PI. The harvested roots around 15 mm were loaded with 20 μM of PI for 15 min at 25°C. The fluorescence was captured using the Olympus FluoView FV1,000 confocal microscope at an excitation of 535 nm and emission of 615 nm.

### Determination of H_2_O_2_ and Malondialdehyde Content

The H_2_O_2_ content was estimated according to the method previously described by Velikova et al. (2000). Root samples (0.25 g) were homogenized with 3 ml of 0.1% (w/v) trichloroacetic acid (TCA) in an ice bath. The supernatant was collected after centrifugation at 12,000 × *g* for 15 min. Next, to the 0.5 ml of 10 mM potassium phosphate buffer (pH 7.0) and 0.75 ml of 1 M KI, 0.5 ml aliquot of the supernatant was added. The H_2_O_2_ content was determined based on the absorbance of the supernatant at 390 nm, and it was expressed as nmol g^–1^ fresh weight. Lipid peroxidation was measured by estimating MDA according to the method of Heath and Packer (1968). Root samples (0.25 g) were homogenized with 5% (w/v) TCA and centrifuged at 11,500 × *g* for 12 min. The supernatant was mixed with 20% TCA containing 0.5% TBA and heated at 95°C for 30 min. The MDA content was measured from the difference in absorbance at 532 and 600 nm, respectively, and expressed as nmol g^–1^ fresh weight.

### Electrolyte Leakage Analysis

The electrolyte leakage (EC) was measured as previously described by Dionisio-Sese and Tobita (1998). Fresh leaf samples (200 mg) were cut into pieces (approximately 5 mm) and placed in test tubes containing 10 ml of deionized water. The test tubes were fixed with plastic caps and incubated in a water bath (32°C). After 2 h, the initial EC of the medium (EC_1_) was quantified using the conductivity meter HI2300 (Hanna Instruments, Woonsocket, RI, United States). The samples were autoclaved at 121°C for 20 min, and the final EC (EC_2_) was measured. Relative EC was calculated by the following formula:


EL(%)=(EC/1EC)2×100.

### Assay of Antioxidant Enzymes

Root samples (100 mg) were frozen in liquid nitrogen and homogenized in 200 μl of different extraction buffer: for ascorbate peroxidase (APX), catalase (CAT) (pH 7.0), peroxidase (POD) (pH 6.0), and superoxide dismutase (SOD) (pH 7.8). The homogenate was centrifuged at 12,000 rpm for 15 min at 4°C. The supernatant was collected to determine the activity of APX, CAT, POD, and SOD. Total proteins were determined with the same buffer solution, and the quantification was made using the Bradford method. The APX activity was quantified according to the decrease in the ascorbate content. The 1-ml reaction mixture contained 830 μl of 50 mM HEPES and 0.1 mM of EDTA buffer (pH 7.0), 50 μl 0.6 mM of ascorbic acid, 100 μl of 0.06 mM H_2_O_2_, and 20μl of crude enzyme solution. The APX activity was recorded at 290 nm, and the results were expressed as μmol g^–1^ fresh weight (Nakano and Asada, 1981). The CAT activity was estimated upon the decrease of H_2_O_2_ content in a 1-ml reaction mixture containing 650 μl of 50 mM PBS (pH 7.0), 330 μl of 0.1% H_2_O_2_, and 20 μl of enzyme solution was prepared. The CAT activity was measured at 240 nm, and the results were expressed as μmol g^–1^ fresh weight (Aebi, 1984). POD was estimated according to the production rate of pyrogallol. The 1-ml of the reaction mixture consists of 420 μl of 100 mM potassium phosphate buffer (pH 6.0), 320 μl of 5% pyrogallol, 6 μl of 30% H_2_O_2_, and 20 μl of enzyme solution. The change was recorded at 420 nm for 20 s after the reaction began and was expressed as μmol g^–1^ fresh weight (Kwak et al., 1995). The SOD activity was detected by determining its ability to inhibit nitroblue tetrazolium (NBT) photochemical reduction. A 1-ml reaction mixture containing 500 μl of 50 mM PBS (pH 7.8), 100 μl and 20 μM of riboflavin, 100 μl and 150 mM of L-methionine, 100 μl and 750 μM of NBT, 100 μl and 100 μM of EDTA, 80 μl of H_2_O, and 20 μl of crude enzyme solution was incubated for 20 min under a white fluorescent lamp. The SOD activity was measured at 560 nm (Beauchamp and Fridovich, 1971).

### 1,1-Diphenyl-2-Picrylhydrazyl Antioxidant Activity Assay

The antioxidant capacity of the samples was measured as previously described by Kumaran and Joel karunakaran (2006) based on the scavenging activity of the stable 1,1-diphenyl-2-picrylhydrazyl (DPPH) free radical. Briefly, various dilutions of the sample extracts were prepared. An aliquot of 500 μl of a diluted extract was added with 500 μl of freshly prepared 0.004% DPPH in methanol and incubated in the dark for 30 min at room temperature. The absorbance at 517 nm against blank was used to calculate [(A_0_–A_1_)/A_0_] × 100, where A_0_ is the absorbance of the control, and A_1_ is the extract.

### Sugar Content Analysis

The glucose content was quantified using a commercial Starch Assay Kit (SA-20, Sigma-Aldrich, St. Louis, MO, United States), and all steps were performed strictly following the technical instruction. The absorbance of samples was measured at 340 nm and expressed as mg-g^–1^dry weight. The sugar content was measured using Agilent 1100 series high-performance liquid chromatography (Agilent Technologies, CA, United States) with the RI -101, refractive index detector (shodex, Denmark). A total of 200 mg of the sample was finely powdered and dissolved in 1 ml of ethanol (80%) and incubated at 50°C for 30 min. Then centrifuged for 3,500 *g* for 15 min and was repeated twice to obtain all the available supernatant. Then the sample was loaded in a metacarp 87 H column and then eluted at 0.5 ml/min at 25°C. The mobile phase consisted of 0.005 M H_2_SO_4_. The standard curve was prepared by using glucose, fructose, and sucrose (mg/ml) at analytical purity. All the samples were repeated and measured three times.

### Number of Apurinic/Apyrimidinic Sites in DNA

A kit obtained from BioVision (Zurich, Switzerland) was used to measure apurinic/apyrimidinic (AP) sites in DNA (three replicates for each treatment group). According to the manufacturer’s instructions, fresh samples were harvested and ground into a fine powder using liquid nitrogen, and DNA was isolated using DNeasy Plant Mini Kit (Qiagen, Hilden, Germany). All genomic DNA samples were diluted to 0.1 μg/μl for accurate assay, and the absorbance of the sample was measured at 650 nm.

### Quantification of Hormones

The quantitative analysis of phytohormones was performed as described by Pan et al. (2010). Fresh root samples of 50 mg were ground into a fine powder using liquid nitrogen and mixed with 500 μl of extraction solvent (2-propanol/H_2_0/concentrated HCL 2:1:0.002, vol/vol/vol), vigorously shaken on a shaking bed for 30 min at 4°C. About 1 ml of dichloromethane was added and again kept in the shaker for 30 min at 4°C. And then centrifuged at 13,000 *g* for 5 min at 4°C. The supernatant was carefully transferred to a new 1.5-ml tube and concentrated using a nitrogen evaporator with nitrogen flow. The sample was redissolved in 0.1 ml methanol, and 50 μl of sample solution was injected into a C18 (reverse-phase) HPLC column for the (HPLC–ESI–MS/MS) analysis. Agilent 1100 HPLC (Agilent Technologies, CA, United States), Waters C18 column (150 × 2.1 mm, 5 μm), and API3000 MSMRM (Applied Biosystems, CA, United States) were used for the analysis. The elution gradient was carried out with a binary solvent system consisting of 0.1% formic acid in water (Solvent A) and 0.1% formic acid in methanol (solvent B) at a 0.5 ml/min flow rate. The content of the hormone (ABA, SA, JA, GA, CTK, and IAA) was determined using a standard external method (ng/g FW) with three biological replications.

### RNA Isolation, Complementary DNA Synthesis, and Quantitative Real-Time PCR Analysis

Total RNA was isolated using the TRIzol reagent (Thermo Fisher Scientific, Waltham, MA, United States), according to the manufacturer’s instructions, and treated with RNase-free DNase I (Promega, WI, United States). The first-strand of complementary DNA (cDNA) was prepared by Revert Aid M-MuLV Reverse Transcriptase (Thermo Fisher Scientific, Waltham, MA, United States) according to the manufacturer’s guidelines. The primers used for quantitative real-time PCR (qRT-PCR) analysis are listed in Supplementary Table 1, Aliquots for qRT-PCR reactions included 20 μl of final volumes containing 1 μl of cDNA, 1 μl of each primer (10 μM), 7 μl of dH_2_O, and 10 μl of SYBR Green PCR Master Mix kit (Toyobo, Japan). All reactions were performed three times in a 96-well reaction plate using the CFX96 Real-Time PCR detection system (Bio-Rad, CA, United States). Three biological replicates were performed for each sample. Ubiquitin gene from the rice was used to normalize, and the change in the transcripts was measured using the 2^–ΔΔ^ CT method.

### Protein Isolation and Immunoblotting Analysis

Immunoblot analysis was performed according to the method of Li et al. (2011). About 200 mg of sample was finely powdered in liquid nitrogen. About 400 μl of aliquot of extraction buffer [62.5 mM TRIS-HCl (pH 7.4), 10% glycerol, 0.1% SDS, 2 mM EDTA, 1 mM phenylmethylsulphonyl fluoride (PMSF), and 5% (v/v) β-mercaptoethanol] was added; then, the sample mixture was vortexed and incubated on ice for 10 min. After two rounds of centrifugation at 12,000 × *g* for 10 min, at 4°C, the supernatants were collected and stored at –80°C until use. The protein concentrations of the samples of the rice tissues were obtained using the Bradford method. For immunoblot analysis, 30 μg of total protein samples were separated on SDS-PAGE 12.5% polyacrylamide gel and transferred to polyvinylidene fluoride (PVDF) membranes. Proteins were determined using a mouse anti-RbcL as a primary antibody (1:5,000; Agri sera AB, Vannas, Sweden) and horseradish peroxidase (HRP) conjugated anti-mouse as a secondary antibody (1:2,000). Using an ECL kit (Bio-Rad, Laboratories, United States), the antibody-protein complexes were visualized.

### Statistical Analysis

The result was shown as the mean of at least three replicates with standard deviation (SD). All the data were analyzed statistically by one-way ANOVA, and the mean differences were evaluated by Fisher’s post-test using the Minitab software (Minitab., Inc., State College, PA, United States). A significance threshold of *p* < 0.05 level was applied.

## Results

### Indole-3-Acetic Acid Improves Plant Growth Under Hydroxyurea-Stress

**FIGURE 1:**
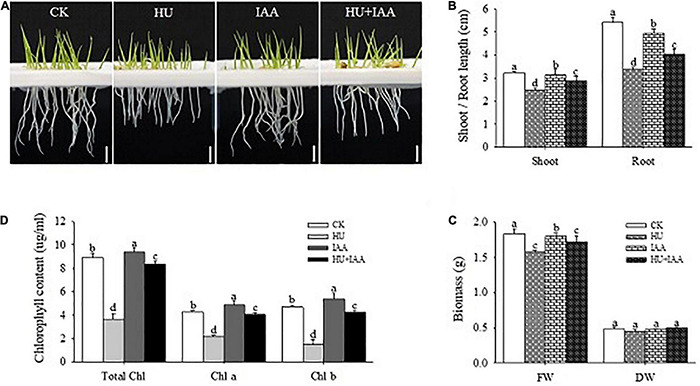
Response of rice seedlings to IAA treatment under HU stress. **(A)** The phenotypic response of 3-day-old rice seedlings to HU, IAA, and their combination treatment. Bar = 1 cm. **(B)** Shoot and root length of the 3-day-old rice seedling, **(C)** plant fresh and dry biomass, and **(D)** chlorophyll contents. Different letters indicate significant differences between treatments, tested by one-way ANOVA followed by Fisher’s test (*n* = 3, *p* < 0.05). CK, Control; HU, Hydroxyurea; IAA, indole-3-acetic acid.

The 3-day-old rice seedlings were treated either with HU or IAA alone and in combination (HU + IAA) for 3 days to know how IAA influenced the HU stress. The results showed that the root length was significantly decreased under HU stress compared to the control, whereas HU + IAA mitigated the root length reduction caused by HU and improved the root length compared to HU-treated plants (Figures 1A,B). Similarly, plants treated with HU + IAA prevented the shoot growth and biomass decrease than the HU-treated plants (Figures 1A–C). Besides, the plants treated with HU significantly reduced chlorophyll content, whereas IAA-treated plants had a slight increase than the control (Figure 1D). When HU and IAA were applied together, chlorophyll content was significantly increased compared to HU-treated plants. Collectively, these results suggested that IAA reduced the effect of HU stress on plant growth.

### Indole-3-Acetic Acid Maintains Photosynthesis Metabolism Under Hydroxyurea Stress

**FIGURE 2:**
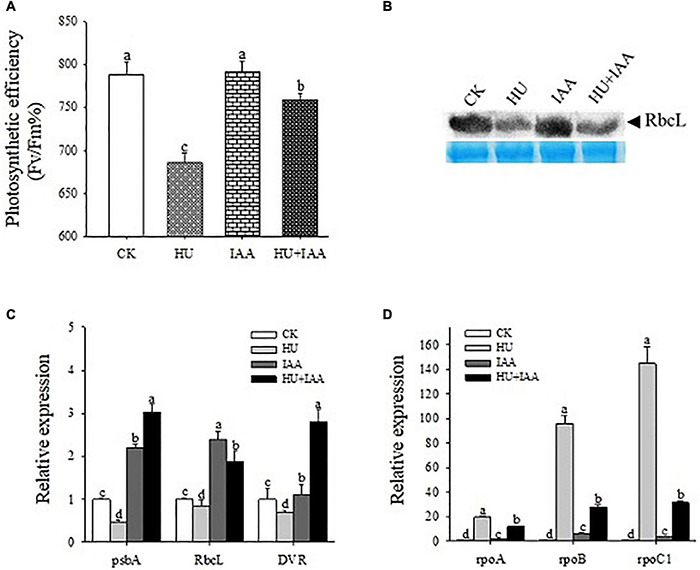
The IAA-treated rice plant’s photosynthesis performance under HU stress. **(A)** Photosynthetic PSII efficiency in the leaves of plants treated with HU, IAA, and their combination (*n* = 8). **(B)** Rubisco large subunit (RbcL) protein expression (top panel) and protein loading control were confirmed by Coomassie blue staining (bottom panel). **(C,D)** Transcript levels of plastid-nuclear genes involved in chlorophyll biosynthesis. Different letters indicate significant changes between treatments, tested by one-way ANOVA followed by Fisher’s test (*n* = 3, *p* < 0.05). CK, Control; HU, Hydroxyurea; IAA, indole-3-acetic acid.

The rice seedlings treated with HU, IAA, and HU + IAA were analyzed for photosynthesis-related parameters to see if IAA maintains photosynthesis performance under HU stress. The photochemical efficiency (Fv/Fm) of Photosystem II was decreased under HU stress compared to the control, while HU + IAA significantly increased the photochemical efficiency of the plants treated with HU (Figure 2A). The photosynthetic efficiency is directly linked to the Ribulose-1,5- bisphosphate carboxylase/oxygenase (Rubisco) enzyme activity. The HU stress significantly downregulated the Rubisco expression in transcript and protein levels compared to the IAA and control. However, it was significantly upregulated by HU + IAA (Figure 2B). Moreover, chloroplast synthesis and development are regulated by the integrated expression of plastid and nuclear genes. Thus, we examined the transcript level of the positive and negative regulators of the genes involved in chloroplast biogenesis. The HU stress suppressed the transcript levels of *psbA*, *RbcL*, and *DVR* genes that are key positive regulators of chloroplast formation and photoautotrophic growth. However, when IAA was combined with HU, the transcript levels of these genes were upregulated (Figure 2C). In contrast, the transcript levels of *rpoA*, *rpoB*, and *rpoC1* genes related to the negative regulation of chloroplast formation were significantly upregulated under HU stress while they were downregulated following HU + IAA treatment (Figure 2D). These observations suggest that HU-induced adverse effects on chloroplast formation and development were enhanced upon HU + I AA treatment.

### Indole-3-Acetic Acid Treatment Increases the Ribonucleotide Reductase Activity and Enhances the Chlorophyll Accumulation

**FIGURE 3:**
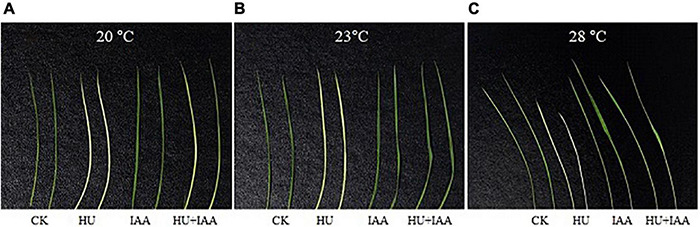
Phenotype characteristics of rice plants treated with HU, IAA, and their combination grown in a plant growth chamber with 12 h of light/12 h of darkness at 20°C, 23°C, and 28°C, respectively **(A–C)**. CK, Control; HU, Hydroxyurea; IAA, indole-3-acetic acid.

As shown in Figures 3A–C, the HU inhibits the activities of the RNRs enzyme that are highly temperature-sensitive. Compared to IAA and control plants, the HU-treated plants showed a chlorosis phenotype in leaves at low temperatures (20 and 23°C). However, this effect was slightly improved under favorable conditions (28°C) compared to control. Notably, HU + IAA-treated plant showed a recovery from chlorosis phenotype at all the temperatures. These results suggest that phenotypic development upon HU + IAA is closely associated with increased RNR activity under ambient temperatures.

### Indole-3-Acetic Acid Treatment Decreases DNA Damage Under Hydroxyurea Stress

**FIGURE 4:**
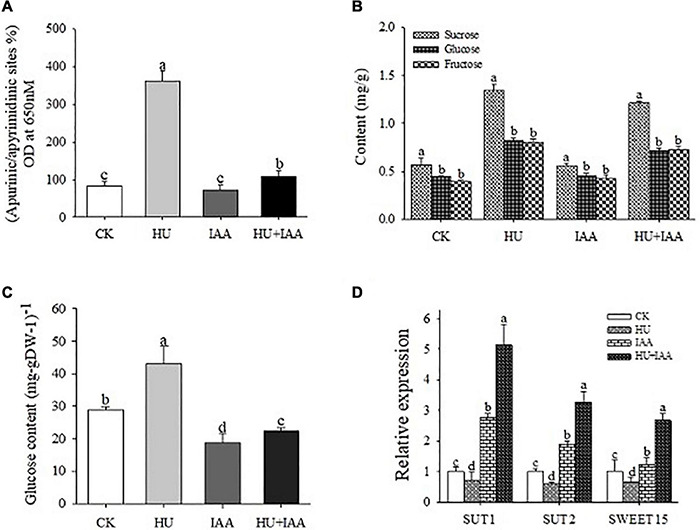
DNA damage in rice plants treated with IAA, HU, and their combination. **(A)** Quantification of apurine/apyrimidine (AP) sites from the plants treated with HU, IAA, and their combination. **(B)** Sugar contents were analyzed using HPLC analysis. **(C)** Glucose contents. **(D)** The transcript level of starch and sucrose transporter genes (*SUT1, SUT2*, and *SWEET15*). Different letters indicate significant differences between treatments, tested by one-way ANOVA followed by Fisher’s test (*n* = 3, *p* < 0.05). CK, Control; HU, Hydroxyurea; IAA, indole-3-acetic acid.

To elucidate the potential role of IAA on the DNA damage induced by HU stress, the HU-induced DNA damage and the resulting alterations in purine/pyrimidine and carbohydrate metabolisms and their transporter activity were evaluated. Interestingly, in the HU-treated plants, the DNA damage in response to H_2_O_2_ was more evident with a substantial upregulation in the accumulation of apurinic/apyrimidinic (AP) sites than the control and IAA-treated plants (Figure 4A). Furthermore, the accumulation of sugar was stimulated in response to HU treatment. Compared to the control and the other treatments, sucrose, glucose, and fructose contents were significantly increased following the HU treatment (Figures 4B,C). Also, the transcript levels of sucrose transporter genes (*SUT1, SUT2*, and *SWEET15*) decreased in HU-treated plants compared to the control (Figure 4D). In contrast, a strong alleviating effect of IAA on HU was observed in HU + IAA treatment; the transcript levels of these genes were increased. It provided significant protection against DNA damage. Overall, these results indicate that IAA alleviated DNA damage and sugar transport inhibition, resulting in the decreased accumulation of sugars in the chloroplast.

### Indole-3-Acetic Acid Regulates the Hydroxyurea-Induced DNA Replication and Cell Cycle Arrest

**FIGURE 5:**
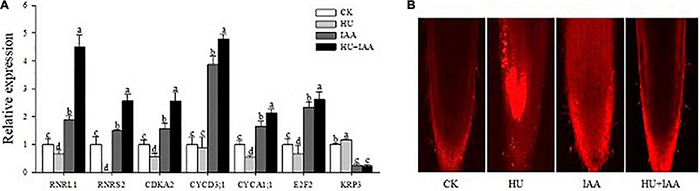
Role of IAA treatment on HU in DNA replication and cell cycle in rice seedlings. **(A)** The transcript level of key cell cycle-related marker genes. **(B)** Confocal images of PI-stained root tips. Different letters indicate significant differences between treatments, tested by the one-way ANOVA followed by Fisher’s test (*n* = 3, *p* < 0.05). CK, Control; HU, Hydroxyurea; IAA, indole-3-acetic acid.

The HU stress is known to disturb the RNR activity that negatively regulates the replication of chloroplast DNA. In contrast, auxin plays a prominent role in regulating both cell proliferation and cell expansion. Thus, to ascertain the specific cell cycle role of IAA in response to HU, we analyzed the transcription level of several genes related to the cell cycle. The *RNR* of large and small subunits (*RNRL1* and *RNRS2*) are related to DNA duplication. Cyclin-dependent kinase (*CDKA2*) primarily expressed in the dividing zone of roots throughout the cell cycle, and their regulatory partner cyclins (*CYCD3; 1* and *CYCA1; 1*) are required for the normal regulation of G1/S and G2/M transitions. Transcription factor 2 (*E*_2_*F*_2_) regulates cell cycle entry and progression in which the transcript levels of all these genes were significantly downregulated in HU treatment relative to the control (Figure 5A). Importantly, IAA and HU + IAA combination treatment displayed a remarkable upregulation of the *RNRs*, *E*_2_*F*_2_, and *CDK-CYCs* genes. In contrast, the CDK inhibitor, *KRP3*, negatively affects cell division, the transcript levels in both IAA and HU + IAA were decreased compared to HU and the control. Furthermore, the accumulation of dead cells triggered by HU treatment was reduced in the HU + IAA treatment (Figure 5B). These results indicate that the addition of IAA alleviated the HU-induced cell cycle arrest by stimulating the RNR.

### Indole-3-Acetic Acid Mitigates the Oxidative Effects of Hydroxyurea Stress by Regulating the Antioxidant System

**FIGURE 6:**
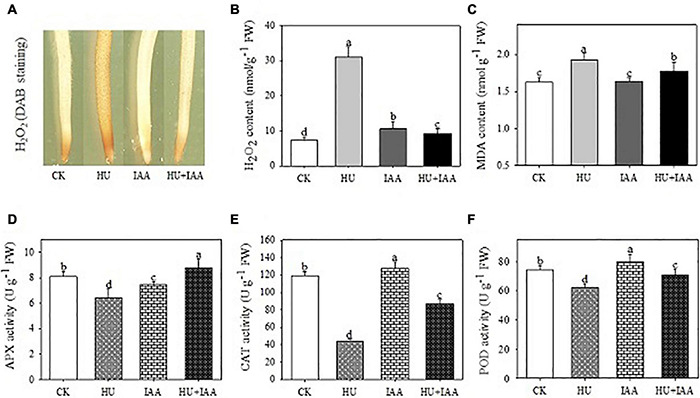
Effect of IAA on physiological and biochemical parameters in the rice seedling exposed to HU stress. **(A)** DAB staining of roots, **(B)** H_2_O_2_ content, **(C)** MDA content, **(D)** APX activity, **(E)** CAT activity, **(F)** POD activity. Different letters indicate significant differences between treatments, tested by the one-way ANOVA followed by Fisher’s test (*n* = 3, *p* < 0.05). CK, Control; HU, Hydroxyurea; IAA, indole-3-acetic acid.

To know the protective role of IAA in plant growth under HU stress, we examined the oxidative stress indicators (H_2_O_2_, MDA, and EL) and antioxidant enzyme activities. The H_2_O_2_ accumulation was visualized through histochemical staining with DAB reagent in root samples, and a solid brown color deposit was observed in the HU than in the control and IAA-treated plants. Furthermore, the H_2_O_2_ content significantly increased under HU stress, which was consistent with qualitative results. On the other hand, plants treated with HU + IAA showed a reduction in brown color deposit and H_2_O_2_ levels compared to HU-treated plants (Figures 6A,B). Likewise, MDA and EL levels were also decreased in HU + IAA-treated plants than in the HU-treated plants (Figure 6C and Supplementary Figure 1A). Moreover, under HU stress, the activities of all the enzymes (SOD, APX, CAT, and POD) were decreased (Figures 6D–F and Supplementary Figure 1B). In particular, the CAT activity was significantly reduced when compared to the control. Conversely, HU + IAA significantly increased the activities of all antioxidant enzymes and their radical scavenging capacity than in the HU-treated plants (Supplementary Figure 4). These results indicated that IAA promoted antioxidant enzyme activity, scavenging the excess H_2_O_2_ accumulated in plant cells under HU stress.

### Replication Stress Role in the Regulation of Indole-3-Acetic Acid-Glucose Conjugation

**FIGURE 7:**
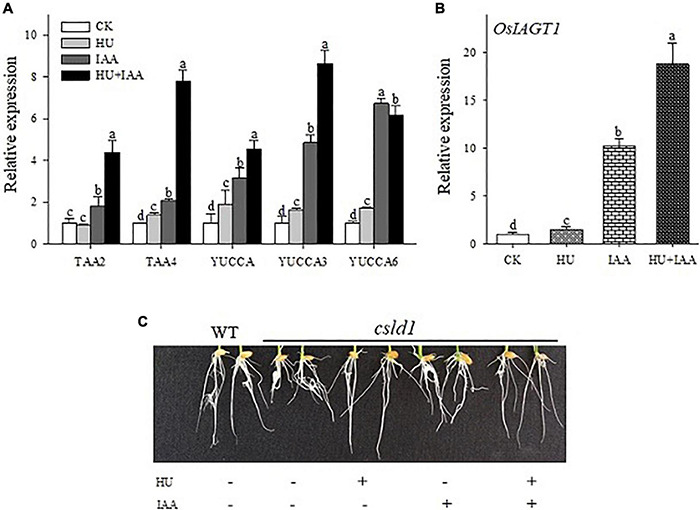
Transcript level analysis of auxin biosynthesis **(A)** and IAA glycosyltransferase (OsIAGT1) marker genes **(B**). Effect of HU, IAA, and their combination treatment on the putative glycosylation *CSLD1*mutant seedling grown for 5 days in a nutrient medium **(C)**. Different letters indicate significant differences between treatments, tested by one-way ANOVA followed by Fisher’s test (*n* = 3, *p* < 0.05). CK, Control; HU, Hydroxyurea; IAA, indole-3-acetic acid.

The inhibition of root growth by HU stress was reversed by HU + IAA treatment. Thus, we examined the role of IAA under HU stress using auxin biosynthesis genes (*TAA2, TAA4, YUCCA, YUCCA3*, and *YUCCA6*). The HU + IAA and IAA treatment showed a significant increase in the transcript levels of all the biosynthetic genes than the control. A slight increase was observed in HU, indicating that hormone levels were altered upon the stress treatment (Figure 7A). In addition, we examined the transcript level of the genes encoding UDP (*UGT90A1*) and auxin-specific (*IAGT1*) glucosyltransferases responsible for mediating the glycosylation reaction. The transcript levels of both the genes (*UGT90A1* and *IAGT1*) were significantly upregulated in HU + IAA than in the other treatments (Figure 7B). Importantly, the auxin transporter gene (*PIN5B*) is vital for exporting and regulating free auxin and their conjugates between the cytoplasm and endoplasmic reticulum (ER). The HU + IAA and IAA displayed a significant upregulation in the transcript level of the *PIN5B* gene (Supplementary Figure 2). This result indicates the transportation of IAA and HU + IAA-induced hormone conjugates from chloroplast to ER. We also investigated the impact of these treatments on the root growth of the putative glycosyltransferase, a *csld1* mutant. Treatment with either HU or HU + IAA promoted the root growth of *csld1* mutant compared to IAA and untreated condition (Figure 7C). Moreover, the transcript levels of cellulose synthase genes (*CSLD4, CesA4*) in HU and HU + IAA were also significantly upregulated compared to IAA and control (Supplementary Figure 2). These results indicated that the sugar accumulation triggered by HU treatment involves hormone conjugation upon HU + IAA treatment. The recovery of glycosylation activity in the *csld1* mutant enhanced the plant growth and development.

### Indole-3-Acetic Acid-Glucose Conjugation Regulates Plant Growth *via* Multiple Hormones Pathway

Plant hormones are essential molecular signals that stimulate plant growth and induce plant tolerance under stress conditions. Therefore, we investigated whether IAA-glucose conjugation is involved in mitigating the plant architecture by triggering the phytohormones. So, we carried out HPLC-MS/MS analysis to quantify the hormone content in the roots under CK, HU, IAA, and HU + IAA treatments. The HU-treated plants showed a significant increase in JA, SA, GA, ABA, and IAA levels compared to the control and IAA-treated plants, with decreasing the CTK levels. While the ABA, SA, CTK, and GA levels were upregulated in HU + IAA compared to all other treatments (Table 1). Transcript-level analysis of SA marker genes (*NPR2, NPR3*, and *WRKY45*) also showed a significant upregulation in HU + IAA than other treatments (Supplementary Figure 3). These results indicate that combined treatment (HU + IAA) induced hormone conjugation, which regulates plant growth by integrating multiple hormone pathways.

**TABLE 1:** Quantification of plant hormones in rice plants treated with IAA, HU, and their combination.

S. no	Hormone (ng/g FW)	Treatment			
		CK	HU	IAA	HU + IAA
1	Abscisic acid (ABA)	3.978 d	6.163 b	5.63 c	16.074 a
2	Salicylic acid (SA)	2.756 c	4.919 b	2.948 c	7.289 a
3	Jasmonic acid (JA)	0.674 c	6.193 a	1.778 c	5.4 b
4	Gibberellic acid (GA)	24.667 d	49.778 b	45.556 c	79.111 a
5	Cytokinin (CTK)	24.222 b	20.519 d	21.385 c	29.481 a
6	Indole-3-acetic acid (IAA)	25.852 b	29.519 a	22.807 d	24.407 c

*The phytohormone contents were analyzed using LC-MS/MS from the roots of the 3-day-old rice seedling treated with HU, IAA, and their combination for 72 h. Different letters indicate significant differences between treatments, tested by one-way ANOVA followed by Fisher’s test (n = 3, p < 0.05). LC-MS/MS, Liquid chromatography–mass spectrometry CK, Control; HU, Hydroxyurea; IAA, indole-3-acetic acid.*

### Indole-3-Acetic Acid-Glucose Conjugation Affects the Auxins Signaling Pathway

**FIGURE 8:**
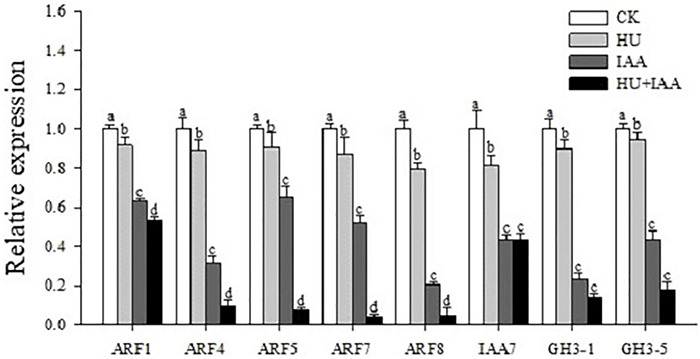
Hormone-glucose conjugation (HU + IAA) negatively regulates the auxin signaling. Transcript analysis of the auxin signaling pathway genes in HU, IAA, and their combination treatment compared to the control. Different letters indicate significant differences between treatments, tested by one-way ANOVA followed by Fisher’s test (*n* = 3, *p* < 0.05). CK, Control; HU, Hydroxyurea; IAA, indole-3-acetic acid.

The phytohormone conjugates do not function as signaling molecules; instead, they fulfill other functions, such as transport and storage. Thus, we performed a transcript level analysis using auxin signaling and downstream marker genes (*OsARF1,OsARF4, OsARF5, OsARF7, OsARF8, OsIAA7, OsGH3-1*, and *OsGH3-5*) to determine the role of IAA-glucose conjugation in auxin signal transduction. The transcript level was significantly downregulated in the IAA-glucose conjugation-induced HU + IAA treatment than the other treatments (Figure 8), which shows that auxin signaling and homeostasis was perturbated in HU + IAA glycosylated plants.

## Discussion

The “interactions” between sugars and phytohormone are regarded as the most important process due to their regulatory role in mediating hormone homeostasis and plant stress tolerance. However, the regulation of signaling and metabolic pathways involved in sugar-hormonal conjugation is still not well understood (Gibson, 2004). The current study shows that IAA forms an ester conjugation with sugar produced from HU-induced DNA damage, resulting in the hormone homeostasis, thus remarkably ameliorating HU-induced toxicity, and enhancing plant growth (i.e., shoot and root) in rice (Figures 1A–D). Chloroplasts are the central hub for metabolic processes, act as environmental stimuli sensors, and synchronize plastid and nucleus-encoded adaptive stress responses. The HU disturbs the RNR activity that severely affects chloroplast biogenesis (Gillard et al., 2008). Recently, the chloroplast localized mutant gene (*slrnrl1*) in tomato has been reported to affect chloroplast division and development (Gu et al., 2020) and the rice virescent3 (v3) and stripe1 (st1) mutants, which encode the large and small subunits of RNR that are sensitive to temperature and important for chlorophyll biogenesis (Yoo et al., 2009). In this study, the transcript level of the plastid genes encoding the transcription/translation apparatus (*rpoA, rpoB*, and *rpoC1*) were significantly upregulated, and the other photosynthetic genes encoded in the plastid (*psbA*) and nucleus (*RbcL*) were downregulated in the HU treatment in comparison to the control. These findings correspond to the studies by Yoo et al. (2009), who reported that the transcript levels of photosynthetic genes encoded in the plastid-nucleus (*psbA* and *LhcpII, RbcL*) were reduced in RNR mutants (V3 and st1). In contrast, the increase in the transcript level was observed in genes encoding transcription/translation apparatus (*rpoA, rpoB*, and *rps7*) compared to the wild type. Ribulose-1,5-bisphosphate carboxylase/oxygenase (Rubisco) is the most abundant enzyme which catalyzes the carbon fixation that is a key for photosynthesis (Bar-On and Milo, 2019). In *Bacillus subtilis*, the HU treatment resulted in the significant downregulation of genes related to carbon uptake due to an insufficient supply of dNTPs for DNA replication and repair (Wozniak and Simmons, 2021). Likewise, in our study, the lower transcript and protein expression trends of the large subunit of Rubisco (*RbcL*) indicated that HU stress reduces the transformation process of carbon assimilation. That disturbs the photosynthetic performance, and the reduction in Fv/Fm displays the decline in PSII efficiency in the HU-treated plants. However, all these changes were mitigated by HU + IAA combined treatment that effectively reversed the HU-induced photosynthetic deficiency (Figures 2A–D). Under ambient temperature, application of IAA to rice seedling root treated with HU stimulates the RNR activity (Figures 3A–C), leading to enhanced chlorophyll accumulation that was significantly reduced by HU stress (Yoo et al., 2009).

Hydroxyurea was previously reported to cause DNA damage through base oxidation and depurination *via* hydrogen peroxide and nitric oxide formation (Sakano et al., 2001). Likewise, the prolonged exposure of HU causes the depletion of the dNTP pool resulting in DNA damage by reducing RNR activity. The purine/pyrimidine and glucose metabolism genes were upregulated, while ribose 5-phosphate and carbon uptake biosynthesis genes were downregulated in the *Bacillus subtilis*. However, the overexpression of RNR genes (*nrdEF*) suppresses the growth inhibition induced by HU, suggesting that RNR is a crucial target of HU (Wozniak and Simmons, 2021). In agreement with the previous literature, in this study, the HU treatment resulted in the oxidative damage of chloroplast DNA, with increased AP site and accumulation of sugar *via* lowering RNR activity (Figures 4A–C, 5A). The significant accumulation of sugar content and its transport inhibition in HU was evident from the transcript level analysis using the sucrose transporters (*SUT1, SUT2*, and *SWEET15*) (Figure 4D). This evidence exhibits that HU caused the high accumulation of sugars in the chloroplast and reduced the photosynthesis gene expression which resulted in the retardation of plant phenotype. Previously, Wang et al. (2018) had reported that the rice glycosyltransferase premature leaf senescence (PLS) mutant displayed a chlorosis phenotype with an elevated level of sucrose and decreased expression of sucrose synthesis and transporters genes than the wild-type plants. Moreover, in this study, the application of IAA and HU decreased the sugars, and purine/pyrimidine accumulation with an increase in sugar transport enhanced plant growth with respect to HU stress. Furthermore, HU + IAA treatment positively regulates cell division. In contrast, the HU reduces the RNR activity resulting in the arrest of chloroplast DNA replication (Cools et al., 2010). Likewise, the tomato RNR mutant (*slrnrl1-1*) gene has been shown to affect the DNA duplication and cell cycle progress. The percentage of *4C* and *8C* cells in *slrnrl1-1* was markedly lower than in the control (Gu et al., 2020). In our transcript level analysis using key cell cycle genes, the HU + IAA treatment displayed the significant upregulation of transcript level in the *CDK-CYCs, RNRs*, and *E*_2_*F*_2_ genes. In contrast, a reduced level of transcripts was observed in the HU-treated sample relative to the control. The transcript level of the cell cycle inhibitor, *KRP3*, was also downregulated in HU + IAA and IAA than in the control (Figure 5A). The Arabidopsis *WEE1*^KO^** mutant displayed a significant accumulation of dead cells triggered by HU treatment, together with N6-benzyl adenine (BA) hormone and HU (BA + HU) treatment, led to the rapid decrease in the vascular cell death phenotype (Cools et al., 2011). Consistent with these previous findings, the PI-stained HU treated roots displayed a significant increase in the accumulation of dead cells, which were substantially reduced in the HU + IAA treatment (Figure 5B). Importantly, antioxidant enzymes act as the first line of defense against ROS in plants. The imbalance between ROS generation and enzymatic detoxification causes oxidative stress that severely affects the membrane integrity (Das and Roychoudhury, 2014). The HU + IAA treatment plays a positive regulatory role in decreasing the membrane lesions triggered by the HU stress. The antioxidant enzyme activities, particularly SOD, CAT, APX, and POD, were enhanced in the HU + IAA treatment with the H_2_O_2_, MDA, and EL content decreased compared to the HU treatment (Figures 6A–F and Supplementary Figures 1A,B). These findings were consistent with the reports of Tognetti et al. (2010), who reported that the overexpression of Arabidopsis auxin hydrogen peroxide–responsive UDP-glucosyltransferase (UGT74E2) results in the hormone homeostasis, followed by an alteration in plant architecture and stress tolerance, by the interplay between H_2_O_2_ and auxin signaling *via* UGT. In addition, the overexpression of Arabidopsis UDP-glycosyltransferase enzyme (OsUGT90A1) plants displayed enhanced antioxidant enzymes to maintain the integrity of the cell membrane against the chilling stress (Shi et al., 2020). The HU has been reported to inhibit ROS (H_2_O_2_) antioxidant scavenging enzymes like CAT and APX in plants (Chen and Asada, 1990; Juul et al., 2010). The increase in CAT and POD activities are interlinked with a decrease in the leaf chlorosis phenotype observed after a growth recovery from ROS-induced stress treatment in rice seedlings (Chen et al., 2012).

The physiological and molecular mechanisms underlying the enhanced seedling growth in response to combination treatment (HU + IAA) were investigated using transcript-level analysis with IAA biosynthesis, UGT, and transporter genes. The IAA can still form ester bonds with sugars catalyzed by UGT following their biosynthesis from the IPA, providing an effective buffer system to maintain cellular auxin content (Wang et al., 2017). Interestingly, the IAA biosynthetic (*TAA2, TAA4, YUCCA, YUCCA3*, and *YUCCA6*) gene transcript levels were upregulated in HU + IAA and IAA treatment than other treatments (Figure 7A) (Ribnicky et al., 1996; Lang et al., 2019). Consistent with our study, the overexpression of the auxin glucosyltransferase gene (*OsIAGT1*) led to the increased transcript level in auxin biosynthesis genes and downregulation of auxin-responsive genes (Liu et al., 2019). The UGT-induced glycosylation modification of IAA plays a critical role in maintaining auxin homeostasis and auxin-related growth activities (Tognetti et al., 2010; Jin et al., 2013). The transcript levels were significantly upregulated in the cytoplasm or chloroplast localized (*UGT90A1*) and auxin-glucose specific (*IAGT1*) UDP- glucosyltransferases genes in the HU + IAA treatment than other treatments (Figure 7B and Supplementary Figure 2). These UGTs are the enzymes that glycosylate a wide range of aglycones, including the plant’s hormones, regulating their bioactivity, solubility, and transport within the cell and throughout the plant (Coutinho et al., 2003; Bowles et al., 2005). In addition, the *PIN5B* transporter gene displayed a high transcript level in HU + IAA and IAA, than in the control (Supplementary Figure 2). ER-localized *PIN5* genes are involved in auxin homeostasis by pumping auxin from the cytoplasm into the ER lumen (Mravec et al., 2009). This is consistent with the previous study that showed *PIN5* overexpression that increases the synthesis of exogenously supplied IAA and its immediate IAA-glucose conversion product in tobacco BY-2 cells. Moreover, the overexpression of *Arabidopsis* thaliana *PIN5* displayed high levels of IAA conjugates than the IAA (Mravec et al., 2009). This evidence suggests that the IAA and HU + IAA-induced auxin conjugates were exported from chloroplast to ER through the PIN transporter gene (*PIN5b*).

Cellulose synthesis is involved in cellulose microfibril biosynthesis, which is essential for cell proliferation and elongation. The putative glycosyltransferases Cellulose Synthase-Like D 1 (CSLD1) mutant has a moderate root sensitivity phenotype (Kim et al., 2007). Surprisingly, upon HU treatment, *csld1* mutant seedling provided concrete evidence about the functional relevance of HU on glycosylation. HU and HU + IAA-treated *csld1* mutant seedling rescued root sensitivity phenotype more significantly than the IAA and control treatment. In addition, HU and HU + IAA displayed high transcript levels in the cellulose synthase (*CSLD4, CesA4*) genes, than in the IAA and control (Figure 7C and Supplementary Figure 2), indicating that sugar supplementation triggered by HU and HU + IAA-induced hormone conjugation recovered the *csld1* mutant root sensitive phenotype. Furthermore, in the previous study, HU treatment has been reported to prevent the UGT1A promoter polymorphisms induced by bilirubin conjugation deficiency, the UGTs enzymes that mediate the conjugation of various compounds with glucuronic acid and facilitate their excretion in the bile. HU treatment on children with UGT1A polymorphism genotype had normal bilirubin levels that prevent gallstone formation (Heeney et al., 2003). The auxin glucosyltransferase (OsIAGLU) gene responsible for regulating root traits was identified using the genome-wide association study (GWAS) approach on rice. The *Osiaglu* mutant displayed reduced root architecture relative to wild type (Zhao et al., 2020), increasing the free ABA, IAA, and JA concentrations and decreasing CTK levels in the roots. Another rice glycosyltransferase (OsUGT90A1) gene protects the plasma membrane from chilling stress by forming sugar conjugates, thereby regulating the plant redox potential *via* the hormone-mediated pathway (Shi et al., 2020). Likewise, our study using HPLC-MS/MS analysis showed an increased level of free JA, SA, GA, ABA, and IAA in HU treatment than the control. The ABA, SA, CTK, and GA were significantly upregulated in HU + IAA (Table 1). Moreover, the transcript analysis using the salicylic acid (*NPR2, NPR3*, and *WRKY45*) displayed remarkable upregulation in HU + IAA than other treatments (Supplementary Figure 3). Therefore, HU + IAA-induced hormone glucose conjugation regulates plant growth through multiple hormone pathways. However, the IAA content was decreased in the HU + IAA treatment when compared to other treatments, which was consistent with those genes involved in auxin signaling. The transcription repressor IAA7, auxin-responsive *ARF1, ARF4, ARF5, ARF7*, and *ARF8* and auxin downstream gene *GH3-1* and *GH3-5* (Wang et al., 2020) displayed the reduced transcript level in HU + IAA treatment than the control treatment (Figure 8). These data were coupled with the earlier finding of Liu et al. (2019) who reported that the overexpression of auxin glucosyltransferase gene (*OsIAGT1*) resulted in the downregulation of auxin content auxin-responsive gene expression. Previously, Topping and Lindsey (1997) observed a considerable lateral root development in the NAA + HU combination treatment, but the resilience mechanism involved in the growth-promoting role remains unclear. Our study is closely in line with these results, but from our obtained results, we elucidate that the protective role of IAA on HU is caused by the glycosylation of hormones that are involved in the modulation of plant growth.

**FIGURE 9:**
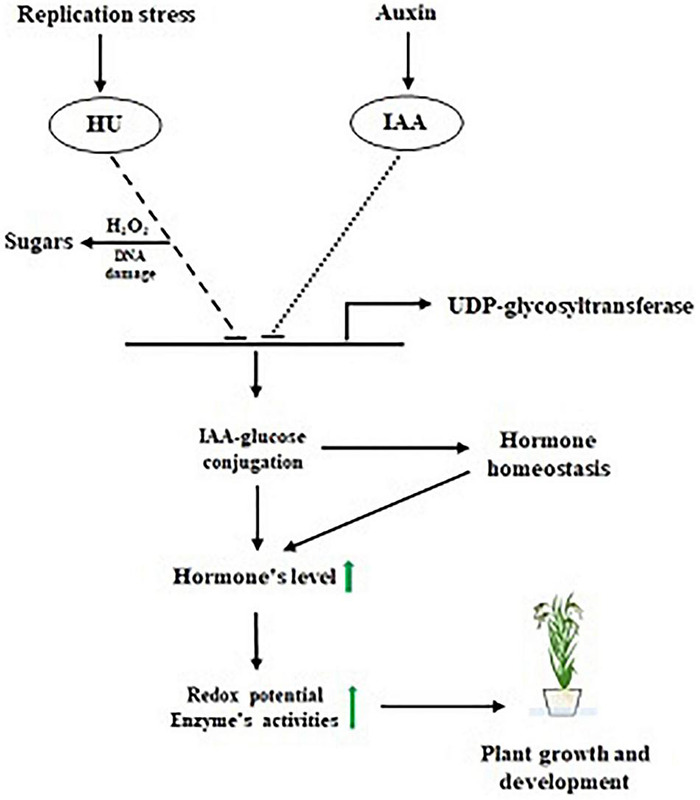
Proposed model for IAA-glucose mediated hormone antagonism.

In summary, we observed that the induction of HU replicative stress, leading to oxidative DNA damage affects the plant architecture. At the same time, the HU + IAA combination treatment-induced hormone-glucose conjugation by (UGT) upregulated the key phytohormones that enhance the plant stress tolerance by decreasing the ROS accumulation (Figure 9). Collectively, our results provide the physiological and molecular evidence of the mitigating effect of IAA against HU-induced phytotoxicity by upregulating the phytohormone and ROS signaling pathway, following hormone-glucose conjugation.

## Data Availability Statement

The original contributions presented in the study are included in the article/Supplementary Material, further inquiries can be directed to the corresponding author/s.

## Author Contributions

YL and VKa conceived and designed this study. VKa, NR, Y-EY, HyC, and K-AL performed the experiments. VKa, NR, MC, Y-NK, HaC, and VKu analyzed the data. VKa, MC, Y-NK wrote the manuscript. All authors contributed to the article and approved the submitted version.

## Conflict of Interest

The authors declare that the research was conducted in the absence of any commercial or financial relationships that could be construed as a potential conflict of interest.

## Publisher’s Note

All claims expressed in this article are solely those of the authors and do not necessarily represent those of their affiliated organizations, or those of the publisher, the editors and the reviewers. Any product that may be evaluated in this article, or claim that may be made by its manufacturer, is not guaranteed or endorsed by the publisher.
