# Supplementary figures and images for: Auxin-Glucose Conjugation Protects the Rice (Oryza sativa L.) Seedlings Against Hydroxyurea-Induced Phytotoxicity by Activating UDP-Glucosyltransferase Enzyme
Source: Front Plant Sci. 2022 Feb 16;12:767044. doi: 10.3389/fpls.2021.767044 (PMC8888425; doi:10.3389/fpls.2021.767044)

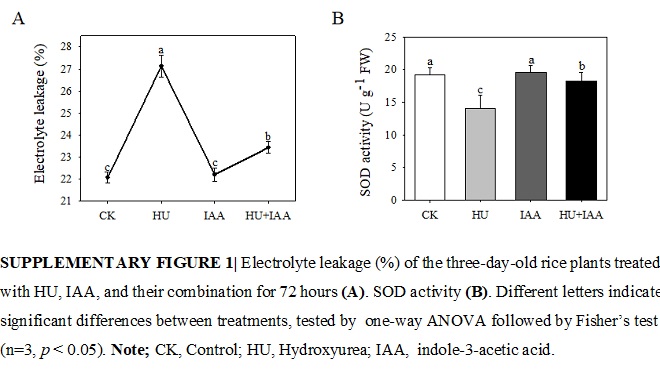

Supplement: Supplementary file 2 [file Image_1.JPEG]

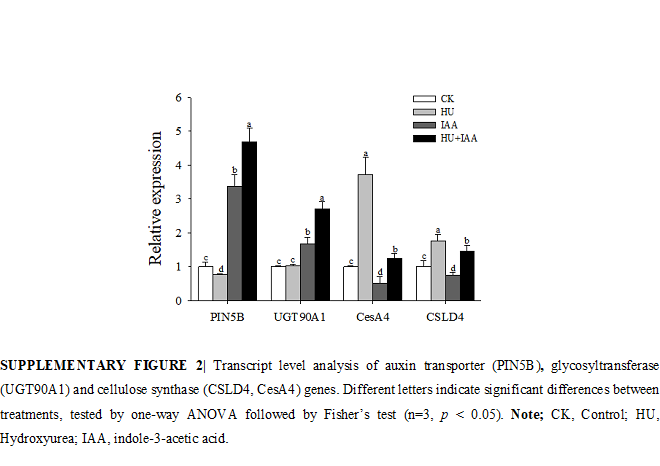

Supplement: Supplementary file 3 [file Image_2.JPEG]

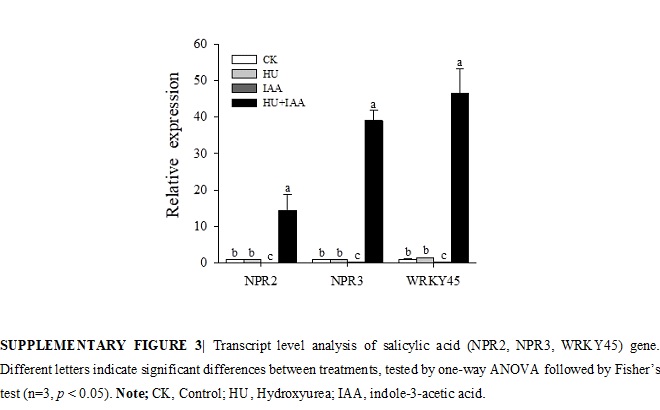

Supplement: Supplementary file 4 [file Image_3.JPEG]

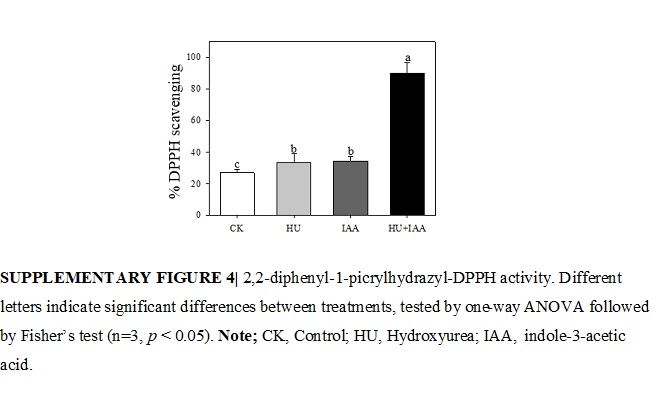

Supplement: Supplementary file 5 [file Image_4.JPEG]
